# Peritoneal mesothelioma in Sweden: A population‐based study

**DOI:** 10.1002/cam4.2436

**Published:** 2019-09-04

**Authors:** Peter H. Cashin, Gabriella Jansson Palmer, Dan Asplund, Wilhelm Graf, Ingvar Syk

**Affiliations:** ^1^ Department of Surgical Sciences, Section of Surgery Uppsala University Uppsala Sweden; ^2^ Department of Molecular Medicin and Surgery Karolinska Institutet Stockholm Sweden; ^3^ Department of Surgery, Institute of Clinical Sciences Sahlgrenska Academy, University of Gothenburg, Sahlgrenska University ‐ Hospital/Östra Göteborg Sweden; ^4^ Department of clinical science Lund University, Skåne University Hospital Malmö Sweden

**Keywords:** cytoreductive surgery, hyperthermic intraperitoneal chemotherapy, Peritoneal mesothelioma, peritoneal metastases, population‐based study

## Abstract

The study aim was to report survival and morbidity of all patients in Sweden with peritoneal mesothelioma treated with cytoreductive surgery (CRS) and hyperthermic intraperitoneal chemotherapy (HIPEC) as well as investigate whether the survival has increased on a population level since this treatment was nationalized 2011. Study data were collected from the Swedish HIPEC registry and the Swedish National Cancer Registry. All patients with peritoneal mesothelioma scheduled for CRS/HIPEC treatment in Sweden January 2011 to March 2018 were retrieved from the Swedish HIPEC registry. Clinicopathological and survival data were collected. For population‐level analysis, all patients with diffuse malignant peritoneal mesothelioma (DMPM) were identified from the Swedish National Cancer Registry and data were retrieved from two separate 5‐year time periods: 1999‐2003 and 2011‐2015. Thirty‐two patients were accepted for CRS/HIPEC. Four were open/close cases. Two‐year survival rate was 84% or 59% when excluding borderline peritoneal mesotheliomas (n = 17). Median overall survival was not reached. Grade III‐IV Clavien‐Dindo events occurred in 22% with no mortality. From the national cancer registry, 102 DMPM cases were retrieved: 40 cases between 1999 and 2003, and 62 cases between 2011 and 2015 (corresponding to an increase from 0.9 to 1.24/million/year, *P* = .04). Six patients (10%) received CRS/HIPEC in the second period. Median OS increased between periods from 7 to 15 months and 5‐year survival from 14% to 29% (*P* = .03). Peritoneal mesothelioma of both borderline and DMPM subtypes undergoing CRS/HIPEC have good long‐term survival. The incidence of DMPM in Sweden has increased. Overall survival has increased alongside the introduction of CRS/HIPEC, which may be a contributing factor.

## Graphical abstract

Peritoneal mesothelioma of both borderline and malignant subtypes can be successfully managed with cytoreductive surgery and hyperthermic intraperitoneal chemotherapy. The incidence of diffuse malignant peritoneal mesothelioma has statistically increased over time in Sweden; fortunately likewise, the overall survival has also increased statistically in part due to the implementation of cytoreductive surgery and hyperthermic intraperitoneal chemotherapy.

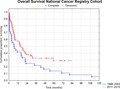

## 1 INTRODUCTION

Cytoreductive surgery (CRS) and hyperthermic intraperitoneal chemotherapy (HIPEC) has been used to treat peritoneal mesothelioma since 1993.^1^, ^2^ A consensus statement from a panel of peritoneal surface oncology experts consider CRS and HIPEC treatment as standard of care for this peritoneal disease.^3^ Due to the rarity of the condition no randomized trial has been performed; however, the reported survival after CRS and HIPEC has exceeded that of systemic chemotherapy in previously published studies, leading to the conclusion that a surgical approach is beneficial. This is in line with basically all current research in the field of peritoneal surface oncology.^4^, ^5^, ^6^, ^7^, ^8^

A number of studies on HIPEC treatment of peritoneal mesothelioma have been published including large multi‐institutional retrospective series,^5^, ^6^, ^7^, ^8^, ^9^ as well as a couple of systematic reviews.^10^, ^11^ In Finland, an epidemiological study was published 2017 demonstrating a significant difference in survival between surgery and chemotherapy (59 vs 9 months). However, no further treatment data were available and the surgical cohort included only nine patients.^12^ In Sweden, peritoneal mesothelioma is an established indication for CRS and HIPEC, and all four Swedish HIPEC centers have developed mutual indications for the treatment of peritoneal mesothelioma. The purpose of this study was twofold: first, to evaluate the outcome of patients with peritoneal mesothelioma undergoing CRS and HIPEC in Sweden from January 2011 to March 2018 using the national HIPEC database; and second, to compare survival on a population level between two time periods, that is, before and after nationalizing HIPEC treatment using the Swedish national cancer registry (1999‐2003 vs 2011‐2015).

## 2 METHODS

For the first aim, data on all patients undergoing CRS and HIPEC due to peritoneal mesothelioma were retrieved from the national HIPEC database between January 2011 and March 2018. The following variables were collected: age, gender, clinicopathological variables, HIPEC variables, morbidity, and survival. For the second aim, data on all patients with a diffuse malignant peritoneal mesothelioma (DMPM) diagnosis were retrieved from the national cancer registry for two separate time periods (1999‐2003 and 2011‐2015), that is, before and after the introduction of national HIPEC treatment guidelines for mesothelioma. The time periods were kept the same size (5 years) for comparison. Borderline peritoneal mesotheliomas, such as cystic and well‐differentiated papillary cases were not available in the National Cancer Registry.

Between 2003 and 2011, occasional patients received CRS and HIPEC at one institution. The number of variables in the National Cancer Registry is limited and the following were available: age, gender, and survival. Subgroup histopathology was only available in the second period. The proportion of patients receiving CRS and HIPEC in the second period was calculated by identifying the patients with DMPM between 2011 and 2015. The study was approved by the county ethical board. The data that support the findings of this study are available on request from the corresponding author. The data are not publicly available due to privacy or ethical restrictions.

### 2.1 Prognostic scores

The prior surgical score is a score that estimates previous surgical trauma related to the peritoneal malignancy being treated. If the PSS is 3 (PSS‐3), ≥5 of the nine abdominopelvic regions (0‐8) have been dissected previously. If two to five abdominopelvic regions have been dissected, it is a PSS‐2. PSS‐1 is for dissection of only one region; which precludes all resections, most commonly for an exploratory laparotomy. PSS‐0 means diagnostic laparoscopy or biopsy only. The completeness of cytoreduction (CC) score denotes the residual disease after completing the CRS part of the surgery. A CC score 0 denotes no visible residual disease. A CC score 1 means there is residual disease up to 2.5 mm in size. A CC score of 2 goes from 2.5 mm to 2.5 cm; and lastly, a CC score 3 means there is bulky disease left >2.5 cm.

### 2.2 Statistics

Descriptive statistics with mean and standard deviations (SD) were used for analysis of demographic data. Fisher's exact test was used to evaluate the incidence increase of malignant mesothelioma over time using Sweden's population of 10 million in the analysis. Survival curves were constructed using the Kaplan‐Meier method. Overall and disease‐free survival was available from the HIPEC registry with March 2018 as the observation cut‐off date. Only crude overall survival was available in the national cancer registry. A two‐tailed log‐rank test was used to compare survival between the first and second time period in the National Cancer Registry cohort.

## 3 RESULTS

### 3.1 National HIPEC registry

**Figure 1:**
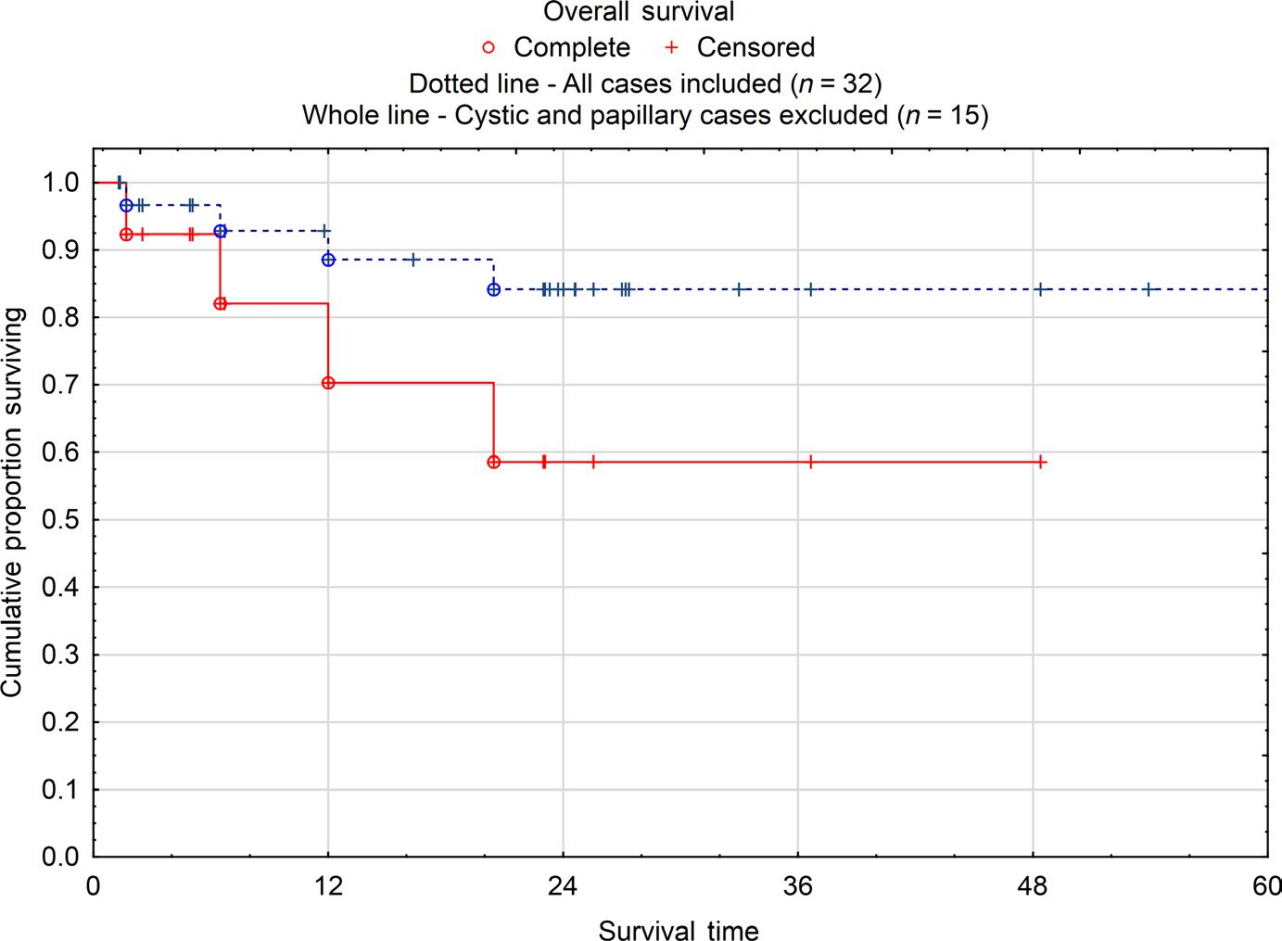
Overall survival of all mesotheliomas in Sweden treated with cytoreductive surgery (CRS) and hyperthermic intraperitoneal chemotherapy (HIPEC) 2011‐2018 and for the malignant subgroup (cystic and papillary mesotheliomas excluded)

A total of 32 patients with peritoneal mesothelioma were accepted for CRS/HIPEC treatment between January 2011 and March 2018. There were 17 borderline subtypes (cystic and well‐differentiated papillary) and 15 DMPM subtypes (Tables 1 and 2). For the whole group, the 2‐year overall survival (OS) of 84% was estimated and the median OS was not reached (Figure 1), whereas the median disease‐free survival (DFS) was 45 months.

**Table 1:** Demographics and tumor characteristics of the national HIPEC cohort 2011‐2018

n = 32	Mean ± SD or n
Age (years)	54 ± 14
Karnofsky performance score	90 ± 10
Gender male/female (n)	13/19
CEA µg/L	1.4 ± 0.9 (1/28 raised CEA > 3.8)
CA 19‐9 kU/L	9.9 ± 14 (1/27 raised CA 19‐9 > 34)
CA 125 kU/L	99 ± 167 (10/28 raised CA 125 > 35)
CA 15‐3 kU/L	42 ± 69 (5/15 raised CA 15‐3 > 25)
C‐reactive protein mg/L	34 ± 72 (10/29 raised CRP > 10)
Platelet count x 10^9^/L	400 ± 365 (7/32 raised Platelet count > 350)
Albumin g/L	34 ± 8 (12/29 lower albumin < 35)
Sarcomatoid	1
Biphasic	4
Epithelial	9
Papillary	9
Cystic	8
Unspecified malignant	1
T stage 1/2/3/4	8/10/9/5
N stage 0/1	31/1
Ki67 low/ high (>10%)/ missing data	4/8/20
Numbers treated	
2011	4
2012	1
2013	2
2014	4
2015	5
2016	8
2017	5
2018 (Jan‐Mar)	3

Abbreviations: CA, Cancer antigen; CEA, carcinoembryonic antigen; CRP, C‐reactive protein; HIPEC, hyperthermic intraperitoneal chemotherapy; SD, standard deviation,

**Table 2:** Treatment related variables in the national HIPEC cohort 2011‐2018

n = 32	Mean ± SD or n
Neoadjuvant chemotherapy for PM (n)	4 (12.5%)
PSS 0/1/2/3 (n)	22/ 5/ 1/ 4
PCI	18.9 ± 10 (Range 2‐39)
Abdominal segments affected out of 9	6 ± 3
Colonic resection (n)	10
Small bowel resection (n)	9
Gastrointestinal anastomosis (n)	5
Diaphragmatic resection (n)	6
HIPEC treatment	25 (78%)
Cisplatinum (+doxorubicin)	17 (14)
50 mg/m^2^ cis/ 100 mg/m^2^ cis	4/ 13
15 mg/m^2^ dox/25 mg/m^2^ dox	5/ 9
Oxaliplatin	7
Mitomycin C	1
No HIPEC (open/close + CRS only)	7 (22%)
HIPEC into the thoracic cavity (n)	4/25 (16%)
Operation time (min)	513 ± 191
Intraoperative bleeding (mL)	1215 ± 1257
CC 0/1/2/open & close (n)	19/7/1/4
Postoperative complications (II‐IVa)	18 (56%)
Clavien‐Dindo II/IIIa/IIIb/ IVa	12/ 4/ 2/ 1 (grade III‐IV 22%)
Adverse events	27 events in 18 patients
Affected kidney function	8
Intraabdominal infection/sepsis	4
Pneumonia/pleural effusion	4
Venous thromboembolic event	3
Postoperative bleeding	1
Strangulated hernia	1
Bowel obstruction	1
Wound dehiscence	1
Thrombocytopenia/ neutropenia	2
Gastroparesis	1
Enterocutaneous fistula	1
Events requiring reoperation (n)	5 (16%)
Adjuvant chemotherapy (n)	7 (22%)

Abbreviations: CC, completeness of cytoreduction score; HIPEC, hyperthermic intraperitoneal chemotherapy; PCI, peritoneal cancer index; PSS, prior surgical score; SD, standard deviation.

**Figure 2:**
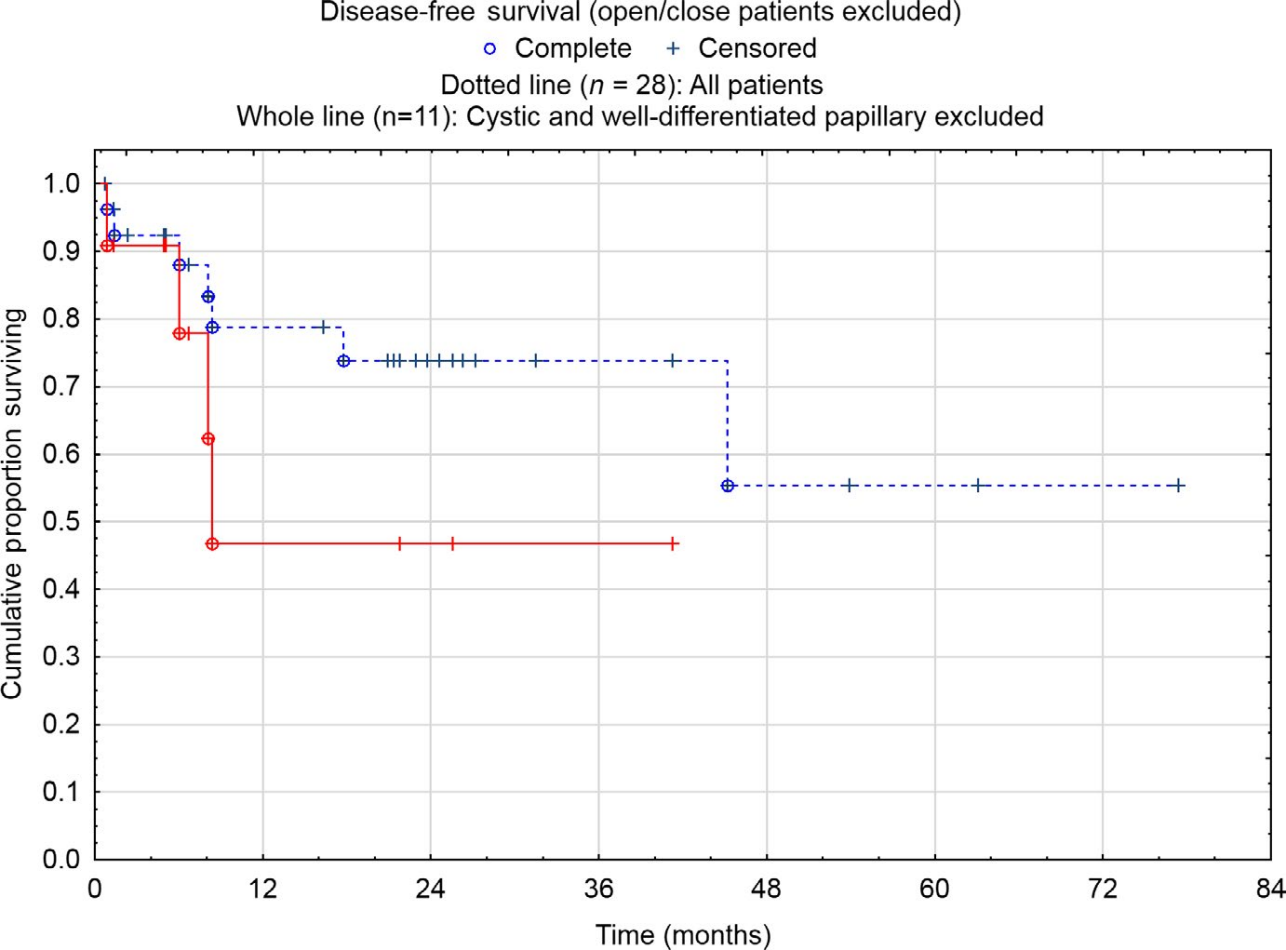
Disease‐free survival of all mesotheliomas in Sweden treated with CRS and HIPEC 2011‐2018 and for the malignant subgroup (cystic and papillary mesotheliomas excluded)

Out of 15 DMPM patients, 11 patients were able to complete CRS/HIPEC. Four of the 11 patients recurred during the study period (median DFS not reached), of which two patients have undergone a second HIPEC treatment. Four patients died during follow‐up, corresponding to a 2‐year OS of 59%. Median OS was not reached (Figure 1), whereas median DFS was 8 months (Figure 2).

Out of the 17 borderline cases, all completed CRS/HIPEC treatment. There were three recurrences (median DFS not reached) and no deaths during follow‐up.

The morbidity according to Clavien‐Dindo was 56% with 22% being grade 3‐4 (Table 2). No in‐hospital mortality occurred. There were two cases of discordant histopathology. One patient had mainly multi‐cystic mesothelioma with a small area in the pelvic region with malignant biphasic type. One patient was initially biphasic, but the recurrence was multi‐cystic borderline.

### 3.2 National cancer registry

**Figure 3:**
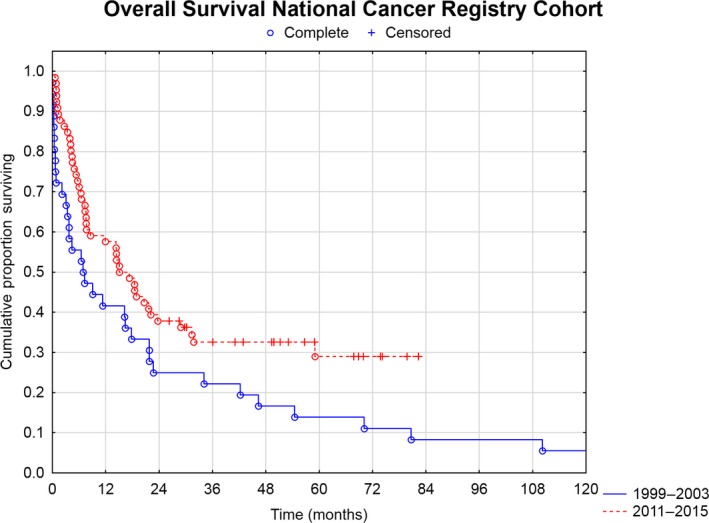
Overall survival of all malignant mesotheliomas in Sweden (autopsy cases excluded) diagnosed 1999‐2003 and 2011‐2015 respectively, that is, before and after the introduction of CRS and HIPEC in Sweden

A total of 102 DMPM patients were identified in the national cancer registry— 40 patients from the first time period (1999‐2003) and 62 patients from the second time period (2011‐2015). A total of six patients (10%) in period two received CRS and HIPEC. The survival doubled between the two time periods with median crude overall survival increasing from 7 to 15 months and 5‐year overall survival increasing from 14% to 29%, *P* = .03 (Figure 3). The median survival of the six patients having undergone CRS+HIPEC in the second time period was not reached but was at least 24 months with the current follow‐up. Moreover, the proportion of patients with DMPM being treat in 2016 and 2017 increased to 6/19 cases (32%), demonstrating that referrals for treatment have increased significantly the last few years. During the 5‐year period from 1999 to 2003, 40 patients developed DMPM amounting to eight patients/year in Sweden or 0.9/million/year. This increased to 1.24/million/year in the second time period (2011‐2015), corresponding to a 38% increase in incidence over 12 years (*P* = .04).

## 4 DISCUSSION

This national population‐based study demonstrates a very good long‐term survival for patients with peritoneal mesothelioma undergoing CRS and HIPEC. This is mainly attributed to the fact that most patients had a borderline histopathology. Well‐differentiated papillary mesothelioma and multi‐cystic cases accounted for about half of the HIPEC cohort. However, even when excluding these patients, the survival plateaued just under 60% after 2 years which is in line with previous studies published.^7^, ^8^ There have been some concerns about treating patients with the sarcomatoid type of DMPM with CRS/HIPEC, but this cohort only included one such patient and no conclusions can be drawn from the study regarding this subtype.

There was no postoperative mortality in this cohort. There were a number of transient acute renal failures attributed to high cisplatin dose (100 mg/m^2^) without thiosulfate renal protection. Four patients underwent concomitant thoracic and abdominal HIPEC following diaphragmatic resection and the average PCI was quite high at almost 19. Despite extensive surgery with PCI 20‐39 (ie, T‐stage 3 to 4 according to a new proposal) 6 in 44% of patients, the survival curves were very encouraging with acceptable morbidity.

Due to the rarity of peritoneal mesothelioma, the knowledge of the natural course of the disease and treatment effects is scarce. In an attempt to evaluate the effect of CRS and HIPEC on the natural history of peritoneal mesothelioma, data from the Swedish national cancer registry were acquired for comparison. Two time periods were chosen: 1999‐2003 and 2011‐2015. The reason that there is a gap between 2003 and 2011 is that there was one HIPEC center in Sweden treating peritoneal mesothelioma with CRS and HIPEC during this time period without a national program in place. From 2011 and onward, a national program for peritoneal surface malignancy was established and four centers were appointed to perform CRS and HIPEC, and peritoneal mesothelioma was an included indication for treatment.

The incidence of DMPM increased during the study period. Over an average 12‐year period, the incidence increased by 38%. The reason for this is unclear, although improved diagnostics as well as an increasing age in the study population may contribute (Table 3). In the first period, there were six patients diagnosed at autopsy, but none in the latter period. This indicates that diagnostics have improved since patients in the latter period were all diagnosed prior to death. Likewise, the main increase in the second period is in the older group (>65). However, the observed rise in incidence needs continued monitoring to determine if it reflects a true increase. While we do not have any epidemiology data on multi‐cystic and well‐differentiated subtypes from the National Cancer registry, as these disease entities are considered borderline and not registered in the cancer registry; it is interesting to note that in the HIPEC registry the incidence is similar between the borderline and malignant subtypes—53% borderline vs 47% DMPM. We have not been able to find any previous publications reporting the proportion between these two groups.

**Table 3:** Demographics of the national cancer registry cohorts: 1999‐2003 and 2011‐2015

n = 102	1999‐2003	2011‐2015
	Mean ± SD (Median: Range) or n (%)	Mean ± SD (Median: Range) or n (%)
Number of patients	40	62
Unexpected autopsy diagnosis	6	0
Male	27	30
Female	13	32
Age 34‐64	20	26
Age 65‐91	20	36
Survival 1 year (excluding autopsy)	15 (44%)	35 (56%)
Survival 2 years (excluding autopsy)	9 (27%)	24 (39%)
Subgroup histopathology		
Biphasic	N/A	3
Epithelial	N/A	14
Malignant unspecified	N/A	45
Number receiving HIPEC treatment	0 (0%)	6 (10%)

Abbreviations: N/A, not available; SD, standard deviation.

The survival of DMPM in the cancer registry has doubled with median survival increasing from 7 to 15 months and 5‐year survival from 14% to 29% (Figure 3). Six (10%) of the 62 patients in the second period received CRS and HIPEC. The median survival of these patients was not reached, but was at least >24 months with the current follow‐up. Thus, their survival has contributed to the survival improvement noted in the latter period. The proportion undergoing CRS and HIPEC was still low despite a national program in place during 2011‐2015; however, when reviewing the incidence data for 2016 and 2017, the proportion of DMPM patients being treated with CRS and HIPEC increased to 32% demonstrating that referrals are increasing for treatment. Data on systemic chemotherapy were not available in the cancer registry making it difficult to ascertain its potential benefit to the population in the second time period. However, looking at the most recent published papers (<10 years old), systemic chemotherapy alone, usually cisplatin and pemetrexed, results in 10‐15 months median OS.^13^, ^14^, ^15^ A first‐line phase 2 study with pemetrexed and gemcitabine reported a 15% response rate and a median OS of 27 months in a highly selected patient group. However, the morbidity was significant (eg, grade 3‐4 neutropenia was 60%) including a mortality of 5% (1/20 patients).^16^ Modest improvements in survival have been made with systemic chemotherapy, which probably also has benefited the patients in the second time period leading to improved outcomes.

The survival of patients with the multi‐cystic and well‐differentiated papillary borderline types of peritoneal mesothelioma operated with CRS/HIPEC was excellent with no deaths yet in the follow‐up, but three recurrences were noted. Multi‐cystic and well‐differentiated papillary types are considered borderline disease entities with a tendency to recur. One of the patients in this study had mainly multi‐cystic mesothelioma with only a small area in the pelvic region with transformation to biphasic malignant mesothelioma (included as a biphasic DMPM patient in the study). As noted in previous research, both multi‐cystic and well‐differentiated papillary types should not be regarded as purely benign diseases but as borderline, where transformation into a malignant disease may occur for both groups.^17^, ^18^, ^19^, ^20^, ^21^ These cases can be treated successfully with CRS and HIPEC.^22^, ^23^, ^24^, ^25^ Unfortunately, the specific role of HIPEC in this setting has not been investigated as it has been introduced together with the CRS approach.

In conclusion, CRS and HIPEC lead to long‐term survival in patients with peritoneal mesothelioma of both borderline and DMPM subtypes with acceptable morbidity and postoperative mortality. The incidence of DMPM may be increasing in Sweden. Survival is also increasing significantly and CRS and HIPEC is probably one contributing factor.

## CONFLICT OF INTEREST

The authors have no conflict to declare.

## AUTHOR CONTRIBUTIONS

Peter Cashin was involved in conceptualization, data curation, formal analysis, funding acquisition, investigation, methodology, project administration, resources, software, validation, visualization, and writing—original draft and editing. Gabriella Jansson Palmer was involved in conceptualization, data curation, investigation, methodology, and writing—review and editing. Dan Asplund was involved in data curation, investigation, methodology, and writing—review and editing. Wilhelm Graf was involved in conceptualization, funding acquisition, investigation, methodology, project administration, resources, and writing—review and editing. Ingvar Syk was involved in conceptualization, data curation, investigation, methodology, project administration, resources, writing—original draft, and writing—review and editing. *Precis (Table of contents)*: Peritoneal mesothelioma of both borderline and malignant subtypes can be successfully managed with CRS and HIPEC. The incidence of malignant peritoneal mesothelioma has statistically increased over time in Sweden; fortunately, the overall survival has also increased statistically in part due to the implementation of CRS and HIPEC.
